# Cost-effectiveness of integrated collaborative care for comorbid major depression in patients with cancer^☆^

**DOI:** 10.1016/j.jpsychores.2015.10.012

**Published:** 2015-12

**Authors:** A. Duarte, J. Walker, S. Walker, G. Richardson, C. Holm Hansen, P. Martin, G. Murray, M. Sculpher, M. Sharpe

**Affiliations:** a Centre for Health Economics, University of York, Heslington, York, UK; b Psychological Medicine Research, University of Oxford Department of Psychiatry, Warneford Hospital, Oxford, UK; c MRC Tropical Epidemiology Group, London School of Hygiene and Tropical Medicine, London, UK; d Robertson Centre for Biostatistics, University of Glasgow, Glasgow, UK; e University of Edinburgh, Centre for Population Health Sciences, Edinburgh, UK

**Keywords:** Collaborative care, Cost-effectiveness, Comorbidity, Depression

## Abstract

**Objectives:**

Comorbid major depression is associated with reduced quality of life and greater use of healthcare resources. A recent randomised trial (SMaRT, Symptom Management Research Trials, Oncology-2) found that a collaborative care treatment programme (Depression Care for People with Cancer, DCPC) was highly effective in treating depression in patients with cancer. This study aims to estimate the cost-effectiveness of DCPC compared with usual care from a health service perspective.

**Methods:**

Costs were estimated using UK national unit cost estimates and health outcomes measured using quality-adjusted life-years (QALYs). Incremental cost-effectiveness of DCPC compared with usual care was calculated and scenario analyses performed to test alternative assumptions on costs and missing data. Uncertainty was characterised using cost-effectiveness acceptability curves. The probability of DCPC being cost-effective was determined using the UK National Institute for Health and Care Excellence's (NICE) cost-effectiveness threshold range of £20,000 to £30,000 per QALY gained.

**Results:**

DCPC cost on average £631 more than usual care per patient, and resulted in a mean gain of 0.066 QALYs, yielding an incremental cost-effectiveness ratio of £9549 per QALY. The probability of DCPC being cost-effective was 0.9 or greater at cost-effectiveness thresholds above £20,000 per QALY for the base case and scenario analyses.

**Conclusions:**

Compared with usual care, DCPC is likely to be cost-effective at the current thresholds used by NICE. This study adds to the weight of evidence that collaborative care treatment models are cost-effective for depression, and provides new evidence regarding their use in specialist medical settings.

## Highlights

• DCPC intervention is clinically effective for the management of depression in cancer patients.
• DCPC vs. usual care in cancer patients is likely cost-effective.
• Findings may impact on decisions on budget allocation in specialist care settings.

## Introduction

Major depression is a leading cause of disability worldwide [1], [2]. It is also an important cause of work place absenteeism and reduced productivity [3]. Major depression that is comorbid with a chronic disease has a particularly large effect: it is associated with substantial decrements in health and a significant increase in patients' use of health care resources [4], [5], [6]. Despite its importance, the treatment of major depression is often suboptimal [7].

The collaborative care model was developed with the aim of improving the management of depression in primary care [8]. The model emphasises systematic treatment delivery and efficient use of specialist skills to deliver evidence-based treatment to a large number of patients. Many trials have found the collaborative care model to be an effective and cost-effective way of treating depression in primary care, and the model is now being developed further to treat depression comorbid with chronic disease [9], [10], [11], [12], [13].

Cancer is becoming a chronic disease for a rapidly increasing number of people [14]. Major depression affects approximately 10% of patients with cancer but, despite the significant health care resources devoted to cancer care, few of these patients receive treatment for depression [15]. ‘Depression Care for People with Cancer’ (DCPC) is a development of the collaborative care model for patients with cancer and comorbid major depression. It is a multicomponent, manualised treatment programme that integrates specialist depression management with both cancer treatment and primary care [16]. The findings of SMaRT (Symptom Management Research Trials) Oncology-2, a 500 patient multicentre randomised controlled trial which found that DCPC was highly effective when compared with usual care, have recently been published [17]. However, its implementation in clinical practice also requires evidence about its cost-effectiveness.

This paper reports on a cost-effectiveness analysis of DCPC compared with usual care from a health service perspective using data from SMaRT Oncology-2.

## Methods

### Study design and participants

SMaRT Oncology-2 was a two-arm, parallel group, multicentre randomised controlled trial in three cancer centres in Scotland, UK (Glasgow, Edinburgh and Dundee) and their associated clinics [17]. The trial included 500 adults (aged ≥ 18 years) with a diagnosis of cancer, a good cancer prognosis (predicted survival ≥ 12 months estimated by their cancer specialist) and major depression (Diagnostic and Statistical Manual of Mental Disorders, 4th Edition [DSM-IV] criteria using the inclusive approach to diagnosis) of at least four weeks' duration [18], [19], [20]. Patients were excluded if they were unable to participate in DCPC (those with substantial cognitive or communication difficulties, or who could not attend regular sessions), or if DCPC was inappropriate to their needs (those with continuous depression for ≥ 2 years, a psychiatric or medical condition requiring alternative treatment, known cerebral metastases, or those already regularly seeing a mental health specialist). Written consent was obtained from all participants. Ethical approval was given by the ‘Scotland A’ Research Ethics Committee (08/MRE00/23).

### Interventions

#### DCPC

DCPC has been described in detail elsewhere [16]. In summary, it is an intensive, manualised, collaborative care-based multicomponent treatment programme specifically designed to be integrated with the patient's cancer treatment. DCPC is systematically delivered by a team that comprises specially trained cancer nurses and supervising psychiatrists working in collaboration with the patient's oncology team and primary care physician. The nurses establish a therapeutic relationship with the patients, provide information about depression and its treatment, deliver brief evidence-based psychological interventions (problem-solving therapy and behavioural activation) and monitor patients' progress. The psychiatrists supervise treatment, aiming to achieve and maintain treatment targets, advise primary care physicians about prescribing antidepressants, and provide direct consultations to patients who are not improving. The initial treatment phase comprises a maximum of ten sessions with the nurse (at the cancer or primary care clinic, or if necessary by telephone) over a four-month period. After this initial treatment period, patients' progress is monitored monthly by telephone (through an automated system supplemented by nurse calls) for a further eight months; additional sessions with the nurse are provided for patients not meeting treatment targets.

#### Usual care

The participant's primary care physician and oncologist were informed about the major depression diagnosis and asked to treat their patients as they normally would. The patient was encouraged to consult their primary care physician to obtain treatment.

### Resource use and costs

The team delivering DCPC recorded: the duration, setting (hospital, home) and professionals (nurse, psychiatrist) present at each treatment session; the duration of all telephone calls to patients and primary care physicians; and related administrative time and average time per patient in supervision sessions. Data were collected on the following healthcare resource use by participant report (using questionnaires administered by post or read out to the patient by telephone interviewers) supplemented by case note review (by clinical researchers to determine the type of appointment, hospital stay or treatment received): inpatient hospital and hospice stays; accident and emergency (A&E) attendances; outpatient appointments for cancer treatment; outpatient appointments for psychological treatment; attendance at NHS-funded day hospices; primary care consultations; relevant prescribed medications (antidepressants, analgesics and anticancer medication). Researchers involved in data collection were blind to treatment allocation.

Total healthcare costs were estimated by multiplying the cost of each unit of resource, using UK national unit cost estimates (pounds sterling at 2010–11 prices), by the amount used [21]. The full cost of training the nurses who delivered DCPC in SMaRT Oncology-2 does not reflect the cost of this training in a real-world setting because nurses will retain the skills acquired for longer than the duration of the trial. Therefore, a more appropriate estimate of this capital cost (as training costs per patient treated with DCPC) was derived by assuming a five-year tenure for each DCPC nurse (with no requirement for re-training), an annual flow of 60 patients per nurse and an annual discount rate of 3.5%. Discounting was not applied to any other costs or outcomes because the time horizon of the study was less than one year.

### Outcomes

Quality-adjusted life years (QALYs) were estimated based on patients' responses to the EQ-5D-3L health-related quality of life (HRQoL) questionnaire at baseline and at 12, 24, 36 and 48 weeks post-randomisation [22]. The EQ-5D-3L asks patients to rate the severity of their problems (no problem, moderate problems or severe problems) in the following domains: mobility, self-care, usual activity, pain/discomfort and anxiety/depression. These ratings define health states which have been assigned scores using preferences measured in a representative sample of the UK population [23], [24]. The EQ-5D scores at each time-point were used to estimate QALYs using the area under the curve method, which multiplies HRQoL weights by time [25]. Mean differences in QALYs were estimated per treatment group using linear regression adjusting for baseline EQ-5D-3L score [26].

### Analysis

A cost-effectiveness analysis was conducted from a healthcare perspective using the intention to treat principle with a time horizon of 48 weeks. The mean difference in healthcare costs incurred and QALYs accrued between treatment groups were estimated using ordinary least squares regression analyses, with robust standard errors to guard against heteroscedasticity [27]. The mean difference in QALYs was adjusted by baseline EQ-5D-3L score to address any baseline imbalance between groups. No other baseline covariate adjustment was performed in the QALY or cost regression analyses for the purpose of this paper. The adjustment of differences in costs and QALYs based on other baseline characteristics (gender, cancer centre, and, cancer type) did not affect the cost-effectiveness results, and regression coefficients were non-significant at a 95% confidence level. These results are, therefore, not shown, but are available on request.

Multiple imputation methods were used with chained equations and predictive mean matching over 10 imputations to estimate cost and EQ-5D-3L data items when these were missing. The following independent covariates were specified for the imputation model: gender, age (≤ 50, 51–60, > 60), baseline EQ-5D-3L score, cancer centre (Glasgow, Edinburgh, Dundee), cancer type (breast, genito-urinary, gynaecological, other).

In the base-case (primary) analysis the additional cost per QALY gained (incremental cost-effectiveness ratio, ICER) of DCPC was calculated compared with usual care based on depression-related healthcare costs (DCPC, antidepressant medication, psychological treatments). This ICER was compared with the cost-effectiveness threshold range of £20,000 to £30,000 per QALY gained (the threshold range adopted by the UK National Institute for Health and Care Excellence [NICE]) [28]. Probabilistic sensitivity analysis was used to estimate decision uncertainty; that is, the probability that the joint uncertainty in costs and QALYs would result in DCPC not being cost-effective at a given cost-effectiveness threshold. These probabilities were presented as cost-effectiveness acceptability curves (CEACs). The analysis was performed by simulating random draws of incremental mean costs and QALYs (n = 1000) from a multivariate normal distribution and estimating the proportion of those draws that corresponded to a cost-effective use of resources at cost-effectiveness threshold values ranging from £0 to £50,000 per additional QALY [29].

Three scenario analyses were conducted to assess the robustness of the findings to alternative assumptions regarding costs and missing data. In principle, only costs that are likely to differ as a result of patients receiving DCPC (rather than usual care) should be included in the analysis. However, the conservative assumption used in the base-case analysis that only depression-related costs would be affected may be incorrect as it is plausible that DCPC could affect the use of other healthcare resources.

In scenario one, the costs associated with inpatient stays, A&E attendances, outpatient appointments, attendance at day hospices and primary care consultations were included, as well as the depression-related healthcare costs used in the base-case analysis. In scenario two, the costs of all resource use collected in the trial were included (by further including the costs associated with analgesics and cancer drugs). In scenario three, only those participants for whom complete data were available were included (using only depression-related healthcare costs).

All analyses were conducted using STATA/SE version 12.0 and Microsoft Excel 2010.

## Results

### Sample characteristics

Of the 500 patients recruited to the trial 253 were allocated to DCPC and 247 to usual care. Participants' characteristics at baseline did not differ between the two groups, except for a slightly longer duration of current depressive episode in the DCPC group. The extent of missing HRQoL and cost data was similar between treatment arms. Further details of the sample have been reported previously [17].

### Resource use and costs

The healthcare resources used over the 48 week follow-up period by trial participants for whom data were available and also the unit costs associated with each type of resource use are shown in Table 1. Resource use was highly variable between individuals, as evidenced by the large standard deviations, but was similar on average between patients allocated to DCPC and usual care, respectively, with the exception of medication use. The proportion of participants prescribed antidepressant medication was higher in those allocated to DCPC than those randomised to usual care (80.4% vs. 59.5%), while the proportion prescribed analgesics was lower (58.67% vs. 67.57%). The amount of missing data was similar between treatment arms for all categories of resource use.

The estimated mean costs of resource use based on imputation of missing data are shown in Table 2. Overall, mean total costs were higher for the DCPC group than for the usual care group (£3463.69 vs. £2924.92 per patient), but the differences in most categories of costs were small. Inpatient stays represented the largest share of healthcare expenditure, comprising over 30% of total costs for both groups. The mean cost of delivering DCPC was £642.13 per patient (representing approximately 19% of the mean total costs incurred by participants allocated to DCPC).

### Outcomes

Table 3 shows the health outcomes at each time point. On average, there was an improvement in HRQoL from baseline until 36 weeks in the DCPC group, but only until 24 weeks for the usual care group. The mean total QALYs accrued during the 48-week trial period were higher for the DCPC group than for the usual care group (0.4913 vs. 0.4413 per patient), (these estimates do not account for differences in baseline HRQoL).

### Cost effectiveness analysis

#### Base-case analysis

**Fig. 1:**
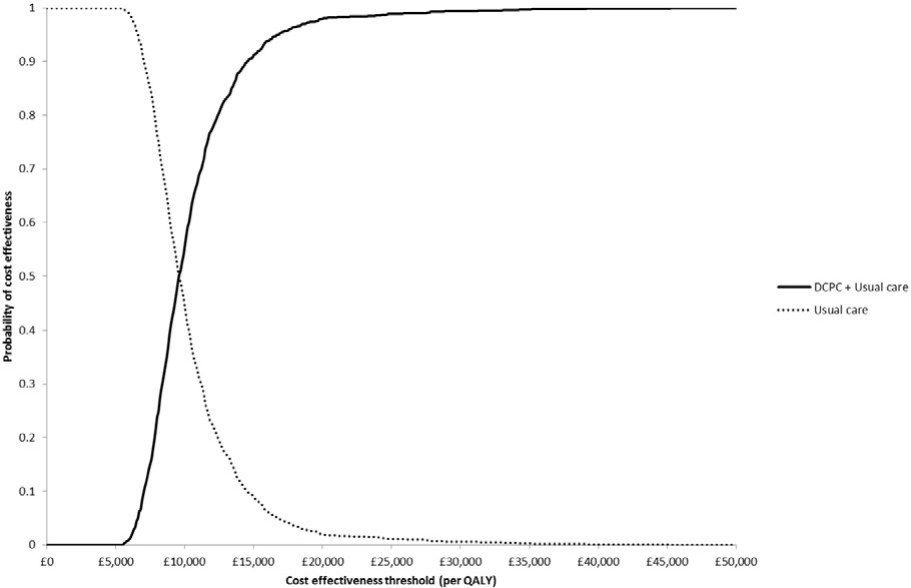
Cost effectiveness acceptability curve for the base-case analysis.

In the base-case analysis DCPC generated 0.066 more QALYs than usual care at an additional cost of £631.30 per patient, yielding an ICER of £9549 per additional QALY (Table 4). The probability of DCPS being cost-effective at the NICE cost-effectiveness threshold range is depicted in Fig. 1. It suggests that, for thresholds above approximately £10,000 per QALY, DCPC is more likely to be the cost-effective treatment than usual care with a probability greater than 0.5. The probability of DCPC being cost-effective when compared with usual care is situated between 0.98 and 0.99 for NICE's commonly adopted range of thresholds (£20,000 to £30,000 per additional QALY).

#### Scenario analyses

The results of the scenario analyses are shown in Table 4. In scenarios one (all collected costs except cancer and analgesic medication) and two (all costs) the findings were consistent with those of the base-case analysis. However, the mean cost difference between DCPC and usual care was smaller in these two scenarios, with a reduction of the corresponding ICERs (£8851.70 and £8149 per additional QALY for scenarios one and two, respectively, compared with the base-case estimate of £9549). Uncertainty was increased by the greater variability of costs (as shown by wider 95% confidence intervals that include zero), and the probability of DCPC being cost-effective decreased but remained higher than 0.9 for the NICE threshold range. The findings of scenario three (based on complete case data) were also consistent with the findings based on imputed data. The mean incremental costs of DCPC compared with usual care were higher than in the base-case analysis (£648 vs. £631 per patient), and the mean incremental QALYs were smaller (0.062 vs.0.066 per patient), resulting in an ICER of £10,400 per additional QALY. The probabilities of DCPC being cost-effective were smaller than in the base case analysis, but remained 0.9 or greater for the considered thresholds. The increased uncertainty in this analysis appears to result from greater variability of both costs and QALYs estimated from the smaller complete case data set (n = 309).

## Discussion

DCPC is likely to be cost-effective when compared with usual care for major depression in patients with cancer. This finding was robust to uncertainty and to the variation of assumptions regarding the types of healthcare costs included and the nature of missing data.

Cost-effectiveness thresholds are used by healthcare systems with limited budgets because, in principle, any new costs incurred by adopting more expensive interventions lead to a reduction in spending and, therefore, health forgone by other types of patient (opportunity costs). The ICER in this cost-effectiveness analysis was £9549 per additional QALY (with a range of £7004 to £10,400 in the different scenario analyses) which is substantially less than the cost-effectiveness thresholds of £20,000 to £30,000 per QALY gained currently used by NICE in the UK [28]. To inform judgements about the NICE cost-effectiveness threshold, recent research has used routine data to provide the first empirical estimate of the health forgone when the NHS incurs additional costs [30]. This suggests that the threshold should be nearer £13,000 per QALY gained. The estimates of the ICER for DCPC in this paper suggest that it remains cost-effective, even at this lower threshold.

We only included depression-related health care use in the base-case analysis to minimise uncertainty and imprecision. However, the scenario analyses, which include other healthcare costs, suggest that DCPC may reduce costs (particularly those not directly related to depression treatment) as well having significant beneficial effects on HRQoL. The incremental costs of DCPC compared with usual care when non depression-related costs are included (£585 and £539 in scenarios 1 and 2, respectively) are always less than the cost of delivering DCPC (£642). This finding should be interpreted cautiously given the relatively small sample size. However it is consistent with the evaluation of DCPC's clinical effectiveness in which patients who received DCPC were found to have much greater improvements not only in depression but also in physical symptoms such as pain [17].

This study has several limitations: First, we relied on self-reported information to obtain data on service use, and supplemented these with data from case note review. Whilst self-report may have led to data inaccuracies, there are no practical alternatives. The accuracy of the cost estimates was also enhanced by the direct recording of all DCPC treatment activities. Second, the analysis is based on the data collected in a single randomised controlled trial and does not, therefore, include all sources of evidence. However, the findings are consistent with those of a previous cost-effectiveness analysis based on data from an earlier efficacy trial of DCPC (n = 200) [31]. A decision analytic model that evaluated the cost-effectiveness of DCPC combined with systematic depression screening for patients attending specialist cancer clinics also suggested that DCPC was likely to be cost-effective (32). Third, the trial was conducted in the one particular health-care system (the UK NHS). However the organisation of cancer care is similar to that in most developed countries with both primary and specialist health-care systems. Fourth, the time horizon of the cost-effectiveness analysis is limited by the duration of the trial (48 weeks) and as costs of the intervention occur early, it is possible that some additional benefit (and, subject to the caveat above, cost savings) would continue beyond 48 weeks. Consequently we may have under-estimated the long-term cost-effectiveness of DCPC.

Published reviews of the cost-effectiveness of collaborative care interventions [11], [33], [34] have, of necessity, focused on studies conducted in primary care settings. This analysis adds to the weight of evidence that collaborative care models are a cost-effective way to deliver depression care and provides new evidence regarding their use in specialist medical settings and for the treatment of depression in cancer patients specifically.

In summary, the collaborative care based DCPC treatment programme is not only an effective treatment for major depression in patients with cancer but also likely to be a cost-effective one. Whist local health care commissioners will need to assess the generalisability of the results of SMaRT Oncology-2 to their own populations, and the appropriate cost-effectiveness threshold in their setting. The results presented here suggest a strong case for the widespread implementation of DCPC into cancer care.

## Declaration of interest

None.

## Figures and Tables

**Table 1:** Healthcare resources used by SMaRT Oncology-2 trial participants and their unit costs over 48 weeks.

Resource	Unit costs			DCPC (n = 253)^a^				Usual care (n = 247)^a^			
Unit	Unit cost or range (£)	Source of unit costs	Mean (SD)	Median (IQR)	Used by (%)	Data completeness (%)	Mean (SD)	Median (IQR)	Used by (%)	Data completeness (%)
DCPC^b^	Contact	44.00–142.50	PSSRU	11.89 (5.35)	13.00 (9–16)	96.0	100.0	–	–	–	–
Psychological treatment	Visits	14.81–68.50	PSSRU	0.27 (1.36)	0 (0–0)	12.6	84.6	1.00 (2.83)	0 (0–0)	18.1	87.4
Antidepressant medication	Item	Various	BNF	–	–	80.4	88.9	–	–	59.5	89.9
Inpatient stays	Length of stay	Various	NHS reference costs	2.2(6.44)	0 (0–1)	25.1	86.6	3.23 (10.49)	0 (0–1)	27.4	88.7
A&E attendances	Visits	117.47	NHS reference costs	0.44(0.79)	0 (0–1)	29.6	87.0	0.32 (0.70)	0 (0–0)	22.2	87.4
GP appointments	Visits	25.00–82.00	PSSRU	10.14 (7.99)	9 (4–14)	93.4	84.2	9.79 (7.66)	9.00 (5–12)	95.8	85.8
Cancer-related appointments	Visits	11.11–118.79	PSSRU NHS reference costs	11.22 (19.06)	5 (2–12)	88.9	78.3	10.36 (15.08)	6 (3–11)	91.1	77.3
Day centres	Visits	28.00	PSS–EX	2.01(8.89)	0 (0–0)	14.2	81.0	2.72 (9.50)	0 (0–0)	17.6	80.6
Cancer medication	Item	Various	BNF	–	–	45.1	88.5	–	–	50.4	89.9
Analgesic medication	Item	Various	BNF	–	–	58.7	88.9	–	–	67.6	89.9

DCPC, Depression Care for People with Cancer; A&E, accident and emergency; BNF, British National Formulary; IQR, interquartile range; PSS-EX, Personal Social Services: Expenditure and Unit Costs; PSSRU, Personal Social Services Research Unit, Unit Costs of Health and Social Care; SD, standard deviation.

a Results based on the available case data set, as multiple imputation was only performed for costs and not for resource use.

b Includes face to face and telephone sessions.

**Table 2:** Unadjusted total costs in pounds sterling of resource use by SMaRT Oncology-2 trial participants (2010–11 prices) over 48 weeks from imputed data sets.

Costs	DCPC (n = 253)			Usual care (n = 247)		
Mean (SE)	95% CI	% total costs	Mean (SE)	95% CI	% total costs
DCPC^a^	642.13 (16.39)	609.85–674.42	18.5	–	–	–
Psychological treatment	7.67 (2.38)	2.99–12.35	0.2	27.28 (4.86)	17.71–36.86	0.9
Antidepressant medication	28.63 (2.16)	24.37–32.89	0.83	19.85 (2.60)	14.73–24.96	0.7
Inpatient stays	1,059.87 (192.45)	680.33–1,439.41	30.6	1,093.57 (231.08)	638.01–1549.14	37.4
A&E attendances	50.56 (6.00)	38.74–62.39	1.5	41.57 (5.45)	30.82–52.31	1.4
GP appointments	367.97 (22.94)	322.75–413.18	10.6	341.94 (19.76)	302.98–380.90	11.7
Cancer-related appointments	542.97 (48.90)	446.59–639.34	15.7	582.45 (43.85)	496.03–668.88	19.9
Day centres	27.95 (7.30)	13.57–42.32	0.8	35.89 (7.36)	21.39–50.39	1.2
Cancer medication	588.30 (171.49)	250.34–926.27	17.0	543.72 (144.17)	259.73–827.71	18.6
Analgesic medication	147.64 (40.18)	68.50–226.77	4.3	238.65 (70.25)	100.28–377.03	8.2
Total	3463.69 (308.40)	2855.74–4071.65	100.0	2924.92 (312.93)	2308.35–3541.52	100.0

a Includes face to face and telephone sessions. A&E, accident and emergency; CI, confidence interval; SE, standard error.

**Table 3:** Unadjusted EQ-5D summary scores and QALYs over 48 weeks from imputed data sets.

Outcome EQ-5D scores	DCPC (n = 253)			Usual care (n = 247)		
Mean (SE)	95% CI	Data completeness^a^ (%)	Mean (SE)	95% CI	Data completeness^a^ (%)
Baseline	0.4492 (0.0196)	0.4107–0.4870	100.0	0.4816 (0.0188)	0.4445–0.5186	100.0
12 weeks	0.5346 (0.0205)	0.4941–0.5751	93.7	0.5016 (0.0196)	0.4630–0.5402	98.4
24 weeks	0.5543 (0.0210)	0.5130–0.5966	93.7	0.4830 (0.0208)	0.4420–0.5240	94.7
36 weeks	0.5566 (0.0212)	0.5148–0.5984	93.3	0.4603 (0.0213)	0.4183–0.5023	96.4
48 weeks	0.5179 (0.0231)	0.4723–0.5635	94.5	0.4534 (0.0225)	0.4092–0.4977	95.1
QALYs	0.4913 (0.0160)	0.4599–0.5228	88.9	0.4413 (0.0155)	0.4108–0.4719	89.9

a In available case data set. CI, confidence interval; SE, standard error.

**Table 4:** Results of incremental cost-effectiveness analysis of DCPC compared with usual care over 48 weeks.

	Data sets used	Costs included	Differences in costs (£)^a^	Differences in QALYs^a^^,^^b^	ICER (£/QALY)	Probability of cost-effectiveness at	
£20,000/QALY	£30,000/QALY
Base case	MI	A	631.30 (595.37; 667.24)	0.066 (0.031–0.101)	9549.16	98.0%	99.4%
Scenario 1	MI	B	585.20 (–78.79; 1249.18)	0.066 (0.031–0.101)	8851.70	92.6%	98.1%
Scenario 2	MI	C	538.76 (–319.34; 1396.85)	0.066 (0.031–0.101)	8,149.30	91.2%	98.7%
Scenario 3	CC	A	648.28 (603.30; 693.26)	0.062 (0.018;0.108)	10,400.25	90.0%	95.6%

A, depression related costs; B, all, except cancer and analgesic medication; C, all costs. CC, complete case data set; ICER, incremental cost effectiveness ratio; MI, multiple imputed data sets.

a Values are mean (95% confidence interval).

b Adjusted for baseline EQ–5D score.
